# Prevalence of virological and serological markers of SARS-CoV-2 infection in the population of Ribeirão Preto, Southeast Brazil: an epidemiological survey

**DOI:** 10.1590/0037-8682-0210-2021

**Published:** 2021-07-02

**Authors:** Edson Zangiacomi Martinez, Afonso Dinis Costa Passos, Amaury Lelis Dal Fabbro, Anderson Soares da Silva, Andreia Cássia Escarso, Antônio Pazin-Filho, Benedito Antônio Lopes da Fonseca, Benedito Carlos Maciel, Daniel Cardoso de Almeida e Araújo, Diego Villa Clé, Gilberto Gambero Gaspar, Jair Lício Ferreira dos Santos, Janise Braga Barros Ferreira, João Paulo Souza, Luane Marques de Mello, Luciane Loures dos Santos, Luzia Márcia Romanholi Passos, Márcio Junio Lima Siconelli, Ricardo de Carvalho Cavalli, Rodrigo de Carvalho Santana, Rodrigo do Tocantins Calado, Sandro Scarpelini, Valdes Roberto Bollela, Vitor Gonçalves Floriano, Fernando Bellissimo-Rodrigues

**Affiliations:** 1 Universidade de São Paulo, Faculdade de Medicina de Ribeirão Preto, Departamento de Medicina Social, Ribeirão Preto, SP, Brasil.; 2 Universidade de São Paulo, Faculdade de Medicina de Ribeirão Preto, Divisões de Imunologia Clínica, Emergência e Doenças Infecciosas, e Unidade de Emergência, Ribeirão Preto, SP, Brasil.; 3 Universidade de São Paulo, Faculdade de Medicina de Ribeirão Preto, Departamento de Clínica Médica, Ribeirão Preto, SP, Brasil.; 4 Universidade de São Paulo, Faculdade de Medicina de Ribeirão Preto, Divisão de Cardiologia do Departamento de Clínica Médica, Ribeirão Preto, SP, Brasil.; 5 Secretaria Municipal de Saúde de Ribeirão Preto, Divisão de Vigilância Epidemiológica, Ribeirão Preto, SP, Brasil.; 6 Universidade de São Paulo, Faculdade de Medicina de Ribeirão Preto, Departamento de Imagens Médicas, Hematologia e Oncologia Clínica, Ribeirão Preto, SP, Brasil.; 7 Universidade de São Paulo, Faculdade de Medicina de Ribeirão Preto, Departamento de Ginecologia e Obstetrícia, Ribeirão Preto, SP, Brasil.; 8 Universidade de São Paulo, Faculdade de Medicina de Ribeirão Preto, Departamento de Cirurgia, Ribeirão Preto, SP, Brasil.; 9 Secretaria Municipal da Saúde, Ribeirão Preto, SP, Brasil.

**Keywords:** SARS-CoV-2, COVID-19, Epidemiological survey, Prevalence, Brazil

## Abstract

**INTRODUCTION:**

This epidemiological household survey aimed to estimate the prevalence of the current and past SARS-CoV-2 infections in Ribeirão Preto, a municipality of southeast Brazil.

**METHODS:**

The survey was conducted in two phases using a clustered sampling scheme. The first phase spanned May 1-3 and involved 709 participants. The second phase spanned June 11-14, 2020, and involved 646 participants.

**RESULTS:**

During the first phase, RT-PCR performed on nasopharyngeal swabs was positive at 0.14%. The serological tests were positive in 1.27% of the patients during the first phase and 2.79% during the second phase. People living in households with more than five members had a prevalence of 10.83% (95%CI: 1.58-74.27) higher than those living alone or with someone other. Considering the proportion of the positive serological test results with sex and age adjustments, approximately 2.37% (95%CI: 1.32-3.42) of the population had been cumulatively infected by mid-June 2020, which is equivalent to 16,670 people (95%CI: 9,267-24,074). Considering that 68 deaths from the disease in the residents of the city had been confirmed as at the date of the second phase of the survey, the infection fatality rate was estimated to be 0.41% (95%CI: 0.28-0.73). Our results suggest that approximately 88% of the cases of SARS-CoV-2 infection at the time of the survey were not reported to the local epidemiological surveillance service.

**CONCLUSIONS:**

The findings of this study provide in-depth knowledge of the COVID-19 pandemic in Brazil and are helpful for the preventive and decision-making policies of public managers.

## INTRODUCTION

Severe acute respiratory syndrome coronavirus 2 (SARS-CoV-2) is the etiological agent of COVID-19, an infectious disease first identified in December 2019 in China before rapidly spreading to all continents^1^ ^,^ ^2^. In March 2020, the World Health Organization (WHO) declared COVID-19 a pandemic^3^ ^,^ ^4^, warning the world of its potentially serious clinical, social, and economic effects. By the end of June 2020, 10,117,687 confirmed cases and 502,278 deaths from the disease had been reported to the WHO, corresponding to an estimated lethality of 4.96% of the known cases confirmed by national epidemiological surveillance systems^4^. COVID-19 spreads predominantly by droplets in the air or fomites, and infection often occurs through contaminated hand contact with the nose, eyes, and mouth. Despite its moderate infectivity, the viral ability to spread may be predominantly attributed to its broad spectrum of clinical manifestations^2^ ^,^ ^5^ ^,^ ^6^. For this reason, social distancing measures have been proposed by several countries to control virus dissemination, in addition to standard infection prevention and control measures such as hand hygiene and wear masks^7^. Social distancing is considered the most effective measure for reducing the spread of SARS-CoV-2 infection, preventing health services from becoming overwhelmed, and, thus, preventing an increase in mortality. Much of the evidence comes from experiences in China, South Korea, Japan, and Singapore, which have adopted restrictions for interpersonal contact^8^. Other countries, such as Italy, Spain, and the United States, have been forced to take extreme measures due to an uncontrolled rise in the cases of infection and imminent risk of healthcare service collapse^8^.

In Brazil, a mathematical modeling study based on data from the São Paulo metropolitan region estimated the outcomes of extended social distancing and no social distancing for two months^9^. In the absence of social distancing, there would be a demand for 5,384 ICU beds (130% of bed capacity), and 1,783 deaths would occur within a month. During the second month, the demand for ICU beds would exceed 14-fold the installed capacity, resulting in an estimated 89,349 deaths. With good adherence to extended social distancing, a maximum of 76% of the total ICU bed capacity would be occupied, resulting in an estimated 317 deaths within the first month and 1,682 deaths within the second month^9^. Although effective, social distancing measures have a significant negative impact on the economy and can worsen the financial and health situation, especially for the poorest and most vulnerable individuals^10^. From a broader perspective, COVID-19 appears to demonstrate a depletion pattern in susceptible people living in clusters, which facilitates a prolonged pandemic phase or a high risk of resurgence in different localities. Knowledge of the prevalence of SARS-CoV-2 infection and its population distribution is important for inferring the effectiveness of social distancing measures and, especially, guiding the gradual and safe reopening of commercial, industrial, educational, and leisure activities. This is especially relevant, considering the risk of underreporting cases due to the large clinical spectrum of the disease^11^ ^-^ ^12^. 

This epidemiological survey aimed to estimate the current and past prevalence of infections due to SARS-CoV-2 in the municipality of Ribeirão Preto (RP) in southeast Brazil at two different time points approximately six weeks apart. The specific objectives were to (a) estimate the seroprevalence of antibodies against SARS-CoV-2 in the RP population, (b) estimate the prevalence of SARS-CoV-2 RNA detection in the RP population, (c) identify the clinical and demographic variables possibly associated with a higher prevalence of current or past infection by SARS-CoV-2, and (d) estimate the infection fatality rate.

## METHODS

### Study area and period

The study area comprises the Ribeirão Preto (RP) municipality, a city in the state of São Paulo (21°10'42″ S latitude and 47°48'24″ W) with an agribusiness-based economy. The population of the municipality was estimated to be 706,552 by 2020, according to the demographic projections of the Brazilian Institute of Geography and Statistics (IBGE)^13^. It was estimated that 12.6% of the population of the municipality were older adults (60 years or older) and 27.2% were 19 years old or younger. 

The data were collected within two periods: May 1-3, 2020, and June 11-14, 2020. According to the official data^14^, 138 confirmed COVID-19 cases and eight related deaths had been reported in RP as of May 3, 2020, and an accumulated 1,973 confirmed cases and 62 deaths had been reported as of June 14, 2020.

### Participants and eligibility criteria

The survey included participants of both sexes and any age living in RP, except neonates, who agreed to participate and signed written informed consent forms. People living in rural areas were excluded; according to the 2010 demographic census data, only 0.28% of the population of RP live in these regions^13^. There were no other exclusion criteria.

### Sampling

The participants were selected by stratified sampling according to the region of residence and social vulnerability. The municipality of RP is divided into five health districts (north, east, west, central, and south). Several census sectors (CSs) were randomly selected from each health district. From these CSs, the households were selected for data collection. The CS is defined as the smallest spatial aggregation unit used by the IBGE to collect socio-economic information from the national census survey. The selection of CSs was based on the São Paulo Social Vulnerability Index, a synthetic indicator proposed by the Brazilian SEADE Foundation to characterize the vulnerability of residents based on income, education, and family life cycle^15^. Only one person was drawn from each household to represent it. The seroprevalence of SARS-CoV-2 was estimated to be less than 20%, with a 95% confidence coefficient and a sampling error of 3.0%. A minimum sample size of 685 people was calculated for the survey. Based on the number of tests available for the research and the possibility of follow-up losses, the final sample size was 709 participants. The number of participants in each health district was proportional to the population size.

### Variables

In this study, the following sociodemographic variables were taken into account: sex, age, ethnicity/color, being a health professional, educational level of the head of the household, monthly family income (in Brazilian currency), the number of persons living in the household, and being an exclusive user of the Brazilian Unified Health System (SUS). Regarding the clinical aspects, the occurrence of the following symptoms at the time of the interview or within the last four weeks before the interview was evaluated: fever, adynamia, myalgia, arthralgia, cough, dyspnea, sore throat, coryza, sternal seizures, anosmia, hyposmia, ageusia, hypogeusia, nausea or vomiting, and diarrhea. The date of appearance of the first symptom and the permanence of symptoms on the day of the interview were also recorded. The primary endpoint of the study was the seroprevalence of total Ig antibodies against SARS-CoV-2 at the two collection timepoints. The secondary endpoint of the study was the frequency of SARS-CoV-2 RNA detection in nasal swab specimens using reverse-transcriptase polymerase chain reaction (PCR).

### Data collection

Before the data collection, the survey was publicized in the local media to foster study participation, and all the data collection team members received training from the survey coordinator. Sampling was performed by considering each of the six regions of the city as a stratum, and the number of people to be interviewed in each stratum was proportional to the total population. To avoid the need for pre-enumeration and ease of fieldwork, a cluster sample was adopted, and a list with 90 index addresses was selected. Each address represented a cluster of eight households, three to the right, and four to the left of the index address. In the case of refusal or the absence of residents, the teams moved to the next house in the series until the required sample was attained. All 50 data collection teams consisted of one interviewer, one phlebotomist, one nasopharyngeal swab collector, and one field supervisor. Each group was responsible for a cluster comprising eight households per day. The teams went to the households, introduced themselves, explained the nature and objectives of the survey, and requested consent to draw one of the household members. The randomization of the household to be included was electronically performed in real-time using a tablet and random selection software. After this selection, the potential participant received a more detailed explanation of the research procedures and was asked to sign an informed consent form.

During the first phase of the study, after signing the consent form, the interviewer administered a questionnaire that included sociodemographic variables. Venous blood was drawn (5mL), and a bilateral nasopharyngeal swab was collected. The participant was asked for a telephone number for contact during the second phase if he or she could not be found at home at that time. The collection of the nasopharyngeal swab was not repeated during the second phase because of the discomfort reported by the participants during the first phase, which could result in a major loss of adherence during the second phase. All procedures were performed by adequately trained professionals wearing appropriate personal protective equipment. The data were collected in tablets and inserted directly into an electronic database using the REDCap platform^16^.

Whole blood samples were processed for the extraction of serum, which was subjected to rapid serological testing (Wondfo®) for the detection of specific antibodies against SARS-CoV-2. The performance evaluation for this serological test conducted by the manufacturer revealed sensitivity and specificity values of 86.4% and 99.6%, respectively^17^ ^-^ ^18^. The nasopharyngeal swabs were subjected to nucleic acid extraction for the detection of SARS-CoV-2 RNA using reverse-transcriptase polymerase chain reaction (RT-PCR). SARS-CoV-2 RNA was isolated from 300 µL of a nasopharyngeal swab suspension. RNA extraction was performed using the Extracta kit AN viral (Loccus) in an automated extractor (EXTRACTA 32, Loccus) following the manufacturer’s guidelines. SARS-CoV-2-RT-PCR was performed using the GeneFinder^TM^ COVID-19 Plus RealAmp Kit (OSang Healthcare Co. Ltd.), which detects the RdRp, E, and N genes^19^. The reaction protocol was performed according to the manufacturer’s protocol using a 7500 Real-Time PCR System (Thermo Fisher Scientific).

### Ethical considerations

The study adhered to the principles of the Declaration of Helsinki and approved by the Ethical Research Committee of the Hospital das Clínicas de Ribeirão Preto (HCFMRP) and the University of São Paulo Ribeirão Preto Medical School (FMRP-USP) (CAAE: 31138820.2.0000.5440). All participants signed an informed consent form before participating in the study. For volunteers younger than 18 years, consent was provided by parents or legal guardians. The study offered minimal health risks to the participants, as the only procedures were data collection and blood analysis. The preliminary results of this survey were immediately communicated to the Hospital Epidemiological Surveillance Center of the HCFMRP and the Epidemiological Surveillance Service of the municipality of RP so that appropriate measures could be taken, including the follow-up of families identified as infected by SARS-CoV-2. This way, participants who tested positive for SARS-CoV-2 infection had the opportunity to receive proper management of their condition. All participants with positive serology results were informed of their diagnosis and its implications on phone. The leftover blood samples were included, with the consent of the participants, in the biorepository "SARS-CoV-2 epidemiological survey in the population of Ribeirão Preto" for further studies. 

### Statistical analysis

The collected data are organized in frequency tables and percentages. The prevalence results were stratified by sex, age group, ethnicity, and other variables of interest. The prevalence ratios (PR) with their associated 95% confidence intervals (95% CI) were used to compare the prevalence of SARS-CoV-2 positivity in the groups. To estimate the percentage of positivity for SARS-CoV-2 adjusted for the distributions of the sex and age groups, we considered the population size projections from the IBGE for 2020. R version 3.6.2 was used to organize the database and perform statistical analyses.

## RESULTS

Overall, 709 participants and 646 (91.1% of the initial sample) were included for the first and second phases of the study, respectively. Twenty-one individuals (3%) refused to participate during the second phase, and 42 (5.9%) were neither found in their homes nor answered phone calls by the data collection teams. Table 1 shows the sociodemographic characteristics of the participants during the first phase of the study, according to the health district of residence. Approximately 56% of the volunteers were women, approximately 64% were Caucasians, and approximately half were between 40 and 69 years old. Only 5.8% were healthcare professionals, and 46% were exclusive SUS users. The differences between the frequencies of sociodemographic characteristics of the different health districts of RP are shown in Table 1; the measure of social vulnerability of each region was considered. Thus, the results in Table 1 show that the sample covers the different social strata of the municipality.

**TABLE 1:** Sociodemographic characteristics of the population sample (n=709) from the 1^st^ phase of the COVID-19 Epidemiological Survey in Ribeirão Preto, according to the health district of residence.

	Health districts					
	Central	East	North	West	South	Total
**Sex**						
Female	70 (59.8)	102 (55.4)	69 (53.5)	101 (54.9)	58 (61.1)	400 (56.4)
Male	47 (40.2)	82 (44.6)	60 (46.5)	83 (45.1)	37 (38.9)	309 (43.6)
**Ethnic background**						
Caucasian	84 (71.8)	142 (77.2)	75 (58.1)	105 (57.1)	47 (49.5)	453 (63.9)
Pardo	21 (17.9)	32 (17.4)	32 (24.8)	55 (29.9)	34 (35.8)	174 (24.5)
Black	10 (8.5)	8 (4.3)	20 (15.5)	21 (11.4)	12 (12.6)	71 (10.0)
Asian	2 (1.7)	1 (0.5)	1 (0.8)	2 (1.1)	1 (1.1)	7 (1.0)
Not declared	-	1 (0.5)	1 (0.8)	1 (0.5)	1 (1.1)	4 (0.6)
**Age range (years)**						
0 - 9	2 (1.7)	6 (3.3)	3 (2.3)	6 (3.3)	4 (4.2)	21 (3.0)
10 - 19	5 (4.3)	13 (7.1)	11 (8.5)	18 (9.8)	15 (15.8)	62 (8.7)
20 - 29	22 (18.8)	16 (8.7)	16 (12.4)	21 (11.4)	5 (5.3)	80 (11.3)
30 - 39	10 (8.5)	24 (13)	16 (12.4)	16 (8.7)	12 (12.6)	78 (11)
40 - 49	18 (15.4)	29 (15.8)	23 (17.8)	29 (15.8)	14 (14.7)	113 (15.9)
50 - 59	18 (15.4)	26 (14.1)	19 (14.7)	35 (19.0)	18 (18.9)	116 (16.4)
60 - 69	22 (18.8)	31 (16.8)	25 (19.4)	27 (14.7)	20 (21.1)	125 (17.6)
70 - 79	10 (8.5)	28 (15.2)	14 (10.9)	22 (12.0)	5 (5.3)	79 (11.1)
80 - 89	7 (6.0)	10 (5.4)	2 (1.6)	7 (3.8)	-	26 (3.7)
90 - 99	3 (2.6)	1 (0.5)	-	3 (1.6)	2 (2.1)	9 (1.3)
**Educational level ofthe head of household**						
Illiterate/incomplete basic education	14 (12.0)	24 (13.0)	46 (35.7)	54 (29.4)	27 (28.5)	165 (23.3)
Complete basic education	12 (10.3)	8 (4.3)	15 (11.6)	24 (13.0)	13 (13.7)	72 (10.2)
Incomplete secondary level	7 (6.0)	4 (2.2)	10 (7.8)	10 (5.4)	6 (6.3)	37 (5.2)
Complete secondary level	30 (25.6)	43 (23.4)	33 (25.6)	59 (32.1)	31 (32.6)	196 (27.6)
Incomplete secondary level	7 (6.0)	14 (7.6)	9 (7.0)	13 (7.1)	1 (1.1)	44 (6.2)
Complete secondary level	29 (24.8)	69 (37.5)	12 (9.3)	19 (10.3)	10 (10.5)	139 (19.6)
Incomplete secondary level	17 (14.5)	20 (10.9)	4 (3.1)	5 (2.7)	6 (6.3)	52 (7.3)
Not declared	1 (0.9)	2 (1.1)	-	-	1 (1.1)	4 (0.6)
**Healthcare professional**						
No	104 (88.9)	171 (92.9)	124 (96.1)	176 (95.7)	93 (97.9)	668 (94.2)
Yes	13 (11.1)	13 (7.1)	5 (3.9)	8 (4.3)	2 (2.1)	41 (5.8)
**Exclusive user of SUS**						
No	80 (68.4)	129 (70.1)	46 (35.7)	87 (47.3)	41 (43.2)	383 (54.0)
Yes	37 (31.6)	55 (29.9)	83 (64.3)	97 (52.7)	54 (56.8)	326 (46.0)
**Number of persons in household**						
1	33 (28.2)	25 (13.6)	14 (10.9)	24 (13.0)	10 (10.5)	106 (15.0)
2	37 (31.6)	59 (32.1)	44 (34.1)	56 (30.4)	26 (27.4)	222 (31.3)
3	21 (17.9)	44 (23.9)	29 (22.5)	47 (25.5)	28 (29.5)	169 (23.8)
4	14 (12.0)	37 (20.1)	27 (20.9)	30 (16.3)	14 (14.7)	122 (17.2)
5	8 (6.8)	15 (8.2)	9 (7.0)	16 (8.7)	8 (8.4)	56 (7.9)
>5	4 (3.4)	3 (1.6)	6 (4.7)	8 (4.3)	9 (9.5)	30 (4.2)
Not declared	-	1 (0.5)	-	3 (1.6)	-	4 (0.6)
**Per capita income** ^a^						
< 500	8 (6.8)	6 (3.3)	29 (22.5)	37 (20.1)	31 (32.6)	111 (15.7)
500 - 1000	26 (22.2)	35 (19)	51 (39.5)	57 (31)	22 (23.2)	191 (26.9)
1000 - 2000	33 (28.2)	62 (33.7)	32 (24.8)	60 (32.6)	28 (29.5)	215 (30.3)
>2000	50 (42.7)	80 (43.5)	17 (13.2)	27 (14.7)	14 (14.7)	188 (26.5)
Not declared	-	1 (0.5)	-	3 (1.6)	-	4 (0.6)

(a) In Brazilian Reals (BRL). In May 4, 2020, 1 US dollar was equivalent to 5.5414 BRL.

Positivity for the RT-PCR test using the nasopharyngeal swab was 0.14% (1/709), and that for the serological test was 1.27% during the first data collection (9/709) and 2.79% (18/646), respectively. No indeterminate results were observed. Table 2 describes some sociodemographic and clinical characteristics of the 22 individuals who tested positive for at least one of the tests during any of the study phases. Of these individuals, 9.1% (2/22) were health professionals, and 31.8% (7/22) reported symptoms.

**TABLE 2:** Sociodemographic and clinical characteristics of the 22 individuals who tested positive for at least one of the tests for SARS-CoV-2 virus infection in Ribeirão Preto, May and June 2020.

Identification	Health district	Health professional	Per capita income	Presence of symptoms	RT-PCR test	Serological test	
						1^st^	2^nd^
RP1023	West	No	4,000	No	Negative	Positive	Positive
RP1024	West	No	1,200	No	Positive	Negative	Negative
RP1117	West	Yes	4,000	No	Negative	Positive	Positive
RP1171	West	No	2,090	Yes	Negative	Positive	Positive
RP2133	East	No	2,500	No	Negative	Negative	Positive
RP2134	East	No	5,000	Yes	Negative	Negative	Positive
RP2149	East	No	11,000	Yes	Negative	Positive	Positive
RP2169	East	No	5,000	No	Negative	Positive	Not found
RP2189	East	No	3,000	No	Negative	Positive	Positive
RP3001	South	No	2,000	Yes	Negative	Negative	Positive
RP3007	South	No	9,000	Yes	Negative	Negative	Positive
RP3052	South	No	3,000	No	Negative	Negative	Positive
RP3093	South	No	3,200	No	Negative	Negative	Positive
RP4031	North	Yes	1,200	No	Negative	Negative	Positive
RP4060	North	No	2,000	No	Negative	Negative	Positive
RP4068	North	No	2,900	No	Negative	Positive	Positive
RP4070	North	No	4,500	No	Negative	Negative	Positive
RP4087	North	No	4,000	No	Negative	Negative	Positive
RP4118	North	No	1,800	Yes	Negative	Negative	Positive
RP5002	Central	No	2,500	No	Negative	Positive	Negative
RP5070	Central	No	2,000	No	Negative	Negative	Positive
RP5131	Central	No	6,500	Yes	Negative	Positive	Positive

Table 3 describes the percentage of positivity detected for the serological or virological markers of SARS-CoV-2 and the respective PR according to the sociodemographic characteristics of the 709 individuals evaluated during the first phase of the study. Considering that the 95% CIs that did not include the null value of 1 provided evidence of differences in prevalence (similar to p<0.05), the results in Table 3 show that family size is associated with test positivity, as families with more than five members had a prevalence that was 10.83 (95%CI: 1.58-74.27) times higher than that found in families with only one or two members. The lack of evidence for an association between the percentage of positivity and the other variables shown in Table 4 is accompanied by fairly wide 95% CIs, a consequence of the few positive cases for the infection.

**TABLE 3:** Percentage of positivity detected for serological or virological marker of SARS-CoV-2 virus infection and respective prevalence ratios (PR) according to sociodemographic characteristics of the 709 individuals evaluated in the 1^st^ phase of the survey. Ribeirão Preto, May 2020.

		Positivity to SARS-CoV-2	
	Total	n (%)	PR (IC95%)
**Sex**			
Female	399	6 (1.5)	Ref.
Male	310	3 (1.0)	0.67 ( 0.17 - 2.64 )
**Health district**			
West	184	3 (1.6)	Ref.
Central	117	2 (1.7)	1.06 ( 0.18 - 6.26 )
East	185	3 (1.6)	1.00 ( 0.20 - 4.89 )
North	129	1 (0.8)	0.50 ( 0.05 - 4.75 )
South	94	0	-
**Ethnic background**			
White	452	7 (1.5)	Ref.
Pardo	175	1 (0.6)	0.4 ( 0.05 - 3.23 )
Black	71	1 (1.4)	0.93 ( 0.12 - 7.47 )
Asian	7	0	-
**Age range (years)**			
0 - 39	241	1 (0.4)	Ref.
40 - 49	114	3 (2.7)	6.75 ( 0.71 - 64.18 )
50 - 59	116	2 (1.7)	4.25 ( 0.39 - 46.39 )
60 - 69	124	2 (1.6)	4.00 ( 0.37 - 43.68 )
70 - 79	79	1 (1.3)	3.25 ( 0.21 - 51.36 )
80 - 99	35	0	-
**Educational level ofthe head of household**			
Illiterate/incomplete basic education	165	3 (1.8)	Ref.
Complete basic education	72	2 (2.8)	1.56 ( 0.27 - 9.11 )
Incomplete secondary level	37	1 (2.7)	1.50 ( 0.16 - 14.02 )
Complete secondary level	196	0	-
Incomplete secondary level	45	1 (2.3)	1.28 ( 0.14 - 11.99 )
Complete secondary level	138	2 (1.4)	0.78 ( 0.13 - 4.59 )
Incomplete secondary level	52	0	-
**Healthcare professional**			
No	668	8 (1.2)	Ref.
Yes	41	1 (2.4)	2.00 ( 0.26 - 15.61 )
**Exclusive user of SUS**			
No	383	6 (1.6)	Ref.
Yes	326	3 (0.9)	0.56 ( 0.14 - 2.23 )
**Number of persons in household**			
1 or 2	328	2 (0.6)	Ref.
3	169	2 (1.2)	2.00 ( 0.28 - 14.07 )
4	122	3 (2.5)	4.17 ( 0.70 - 24.64 )
5	56	0	-
>5	31	2 (6.5)	10.83 ( 1.58 - 74.27 )
**Per capita income** ^a^			
<500	112	1 (0.9)	Ref.
500 a 1000	191	3 (1.6)	1.78 ( 0.19 - 16.89 )
1000 a 2000	215	4 (1.9)	2.11 ( 0.24 - 18.66 )
>2000	188	1 (0.5)	0.56 ( 0.04 - 8.79 )

(a) In Brazilian Reals (BRL). In May 4, 2020, 1 US dollar was equivalent to 5.5414 BRL.

Based on the percentage of positivity for the virologic test (RT-PCR) adjusted for the distributions of sex and age group, we estimated that approximately 0.11% (95% CI: 0.08 - 0.14) of the RP population were actively infected with SARS-CoV-2 during the first phase, which is equivalent to 755 individuals (95% CI: 558-952) (Table 4). Considering the percentage of positive results for the serological tests, also adjusted for sex and age group distributions, we estimated that approximately 2.37% (95% CI:1.32-3.42) of the population would be cumulatively infected with SARS-CoV-2 by mid-June 2020, which is equivalent to a total of 16,745 residents (95% CI: 9,326-24,164) (Table 4). Considering the estimated prevalence for the past infections and the 62 COVID-19-related deaths confirmed among residents by the time of the second phase of the survey (based on official data^12^), the local COVID-19 fatality rate may be estimated at 0.37% (95% CI: 0.25-0.67), which is equivalent to approximately four deaths for every 1,000 individuals infected with the virus. If we consider that there were 1,973 reported and confirmed cases of COVID-19 among residents of the municipality up to the date of the second phase of the survey (based on official data^14^), we can estimate that only 11.78% (1,973/16,745) of the cases of SARS-CoV-2 infection were reported by the local health services, which led to the estimation of the existence of at least 8.5 asymptomatic or subclinical patients for each case reported by the epidemiological surveillance system.

**TABLE 4:** Estimated prevalence of active and past infection by SARS-CoV-2 and corresponding estimated number of infected persons in the population of Ribeirão Preto, in the two phases of the survey, respectively in May and June 2020.

	Estimated prevalence (95%CI)	Estimated number of infected persons^a^ (95%CI)
**Active SARS-CoV-2 infection (1** ^st^ **phase)**	0.11% (0.08 - 0.14)	777 (565 - 989)
**Past SARS-CoV-2 infection (1** ^st^ **phase)**	1.10% (0.35 - 1.86)	7,772 (2,473 - 13,142)
**Past SARS-CoV-2 infection (2** ^st^ **phase)**	2.37% (1.32 - 3.42)	16,745 (9,326 - 24,164)

(a) Based on a population of 706,552 inhabitants.

## DISCUSSION

In this study, we showed that the RT-PCR positivity was 0.14% for the nasopharyngeal swabs and 1.27% and 2.79% for the serological tests performed during the first and second phases of the survey, respectively. Therefore, individuals tested positive for at least one of the tests during any of the study phases. Only 31.82% (7/22) of these individuals with laboratory evidence of SARS-CoV-2 infection had one or more symptoms consistent with COVID-19 up to 30 days before the tests were collected. One person with a positive RT-PCR result denied any clinical symptoms related to the disease at the time of sampling. Two days after collection, he/she was personally reassessed by the technical team and was still asymptomatic. The entire family of this person was clinically evaluated and underwent laboratory tests, and three other persons tested positive for SARS-CoV-2 on RT-PCR; one was asymptomatic and the other two were oligosymptomatic. The entire family of this person was instructed to remain in vertical isolation for 14 days and practice strict personal hygiene. Two of the individuals who tested positive on the serology evaluation during the first survey tested negative during the second survey. This confirms what has been found by other authors regarding the potential low durability of the humoral immunization induced by SARS-CoV-2 infection, raising the possibility of reinfection^20^ ^-^ ^21^.

The present population-based survey detected a relatively low prevalence of active and past SARS-CoV-2 infections in the RP population during May and June 2020. After adjustments for sex and age, we estimated that approximately 2.37% of the RP population had been cumulatively infected by SARS-CoV-2 in mid-June 2020. We also estimated that approximately 0.11% of the population was actively infected with SARS-CoV-2 during the first phase of the study. These numbers are in accordance with those reported in other Brazilian serological household surveys. In a study of IgG antibodies against SARS-CoV-2 by serology (ELISA) in samples of the adult population of São Carlos, a municipality near RP, four repeated cross-sectional surveys were carried out, each evaluating 1,400 individuals with an interval of 15 days between them^22^. Among the participants, 13 (1.2%) and 32 (2.7%) tested positive during the first and fourth phases, respectively. A limitation of this study is that it did not provide data collection dates to allow for direct comparisons. In a population-based survey in southern Brazil, using the Wondfo® lateral flow point-of-care test for immunoglobulin M and G antibodies against SARS-CoV-2, the seroprevalence was 0.048% for April 11-13 (round 1), 0.135% for April 25-27 (round 2), and 0.222% for May 9-11 (round 3), with a significant upward trend over the course of the surveys^23^. A large cross-sectional study including two seroprevalence surveys in 133 sentinel cities in all Brazilian states and the Wondfo®SARS-CoV-2 Antibody Test showed a seroprevalence adjusted for sampling design and test validities of 0.4% and 0.6% for the Southeast region during the first and second surveys (May 14-21 and June 4-7, 2020), respectively^24^. 

On the other hand, the seroprevalence estimated for the RP population appears to be lower than that reported by other studies conducted in other countries. For instance, in a survey carried out in Spain at the national level between April 27 and May 11, 2020, the seroprevalence was 5.0% for the point-of-care test and 4.6% for immunoassay^25^. This study showed substantial geographical variability, with a prevalence that was higher around Madrid (>10%) and lower in coastal areas (<3%)^25^. In April 2020, the prevalence of antibody seropositivity was estimated to be 22% in the Guilan province in Iran^26^ and 3.1% in Geneva in Switzerland^27^. In general, a systematic review and meta-analysis published in March 2021, which included 47 studies, showed that the SARS-CoV-2 seroprevalence in the general population of 23 countries varied from 0.37% to 22.1%, with a pooled estimate of 3.38%^28^. Another study not included in this systematic review showed that the seroprevalence in the population of the Lambayeque region located in the north of Peru was 29.5% (data collected from June to July 2020)^29^. The authors claimed that Lambayeque was the region with the highest seroprevalence of SARS-COV-2 globally at the time of the research^29^. Even so, the number of moderate and severe cases reported in RP in 2020 was enough to saturate the hospital health system, which required new hospital beds for both the public and private systems.

**FIGURE 1:**
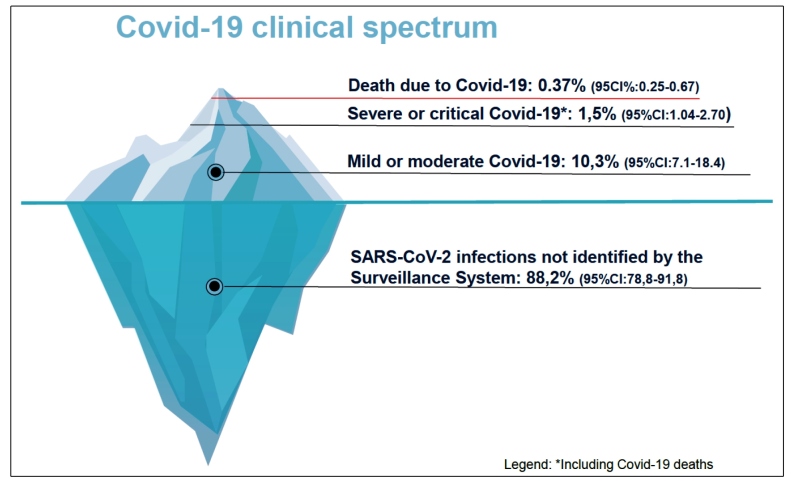
Proportion of reported and confirmed cases of Covid-19, according to clinical presentation, in relation to the number of SARS-CoV-2 infections estimated by survey data.

Comparing the data from this survey with the official data available from the Epidemiological Surveillance Service of the municipality allows for a better understanding of the clinical spectrum of COVID-19. A positive aspect of this comparison is that the estimated infection fatality rate (0.37%) is significantly lower than the official case fatality rate (CFR) for RP calculated at 3.14% (62/1,973). On April 29, 2020, the CFR for Brazil was estimated at 6.94 (95%CI: 6.76 - 7.13)^30^. However, approximately 88% of the cases of SARS-CoV-2 infection are not reported to the local epidemiological surveillance service, and this is worrisome because it is assumed that the majority of those infected are either asymptomatic or oligosymptomatic, which makes it extremely difficult to control the disease at the population level. These people tend to continue their daily routine and may have innumerous opportunities to transmit the virus to susceptible people during social interactions or in the workplace^2^ ^,^ ^5^ ^,^ ^6^. Asymptomatic or oligosymptomatic persons have a lower individual capacity for disease transmission; however, at the population level, they account for the majority of transmissions, given their high frequency of occurrence. This assumption underscores the importance of general prevention and control measures, such as social distancing, wearing a face mask, and hand hygiene when compared with measures focused on the vertical isolation of confirmed cases. Although all of them are necessary, it is reasonable to assume that the former are more effective than the latter, which is gradually being confirmed in the literature^31^ ^-^ ^33^. The pictogram in Figure 1 shows the proportion of reported and confirmed cases of COVID-19, according to the clinical presentation, in comparison to the number of SARS-CoV-2 infections estimated by the survey data.

The strengths of the study include the use of a broad and representative sampling approach, considering the heterogeneity of the population of RP based on social indicators^34^, and the use of tests with the ability to detect past and active infections. Among its limitations, it was impossible to adjust the prevalence estimates of the accuracy of the tests by adequate methods^35^ because of the few individuals who tested positive. The use of logistic regression models to obtain PR measures adjusted for potential confounders or models based on complex sampling was also not possible for this reason. The sample size was determined based on a sampling error of 3.0% for estimating the percentage of positivity for the virologic test, and it was limited by the available number of tests for SARS-CoV-2 infection. The number of participants was sufficient to estimate the seroprevalence of the infection with reasonable precision, but the confidence interval for the population that had been cumulatively infected was quite wide (95%CI: 9,267-24,074 inhabitants). Additionally, the relatively low sensitivity of the serological test (86.4%) may have underestimated the true prevalence of past infections. Despite these problems, these results may contribute to the technical and political planning of actions to confront the crisis due to COVID-19 that, at the time of this writing, was still responsible for a high number of cases and deaths in RP and other cities in the region.
